# Z‐Scheme Photocatalytic Systems for Promoting Photocatalytic Performance: Recent Progress and Future Challenges

**DOI:** 10.1002/advs.201500389

**Published:** 2016-04-13

**Authors:** Haijin Li, Wenguang Tu, Yong Zhou, Zhigang Zou

**Affiliations:** ^1^ Key Laboratory of Modern Acoustics (MOE)Institute of Acoustics, Department of PhysicsNanjing UniversityNanjing210093JiangsuP. R. China; ^2^ School of Mathematics and PhysicsInstitute of Optoelectronic Information Materials and TechnologyAnhui University of TechnologyMa'anshan243002AnhuiP. R. China; ^3^ National Laboratory of Solid State MicrostructuresCollaborative Innovation Center of Advanced Microstructures School of PhysicsJiangsu Key Laboratory for Nano TechnologyNanjing University22 Hankou RoadNanjingJiangsu210093P. R. China

**Keywords:** charge carriers, connection modes, electron mediators, photocatalysts, Z‐scheme

## Abstract

Semiconductor photocatalysts have attracted increased attention due to their great potential for solving energy and environmental problems. The formation of Z‐scheme photocatalytic systems that mimic natural photosynthesis is a promising strategy to improve photocatalytic activity that is superior to single component photocatalysts. The connection between photosystem I (PS I) and photosystem II (PS II) are crucial for constructing efficient Z‐scheme photocatalytic systems using two photocatalysts (PS I and PS II). The present review concisely summarizes and highlights recent state‐of‐the‐art accomplishments of Z‐scheme photocatalytic systems with diverse connection modes, including i) with shuttle redox mediators, ii) without electron mediators, and iii) with solid‐state electron mediators, which effectively increase visible‐light absorption, promote the separation and transportation of photoinduced charge carriers, and thus enhance the photocatalytic efficiency. The challenges and prospects for future development of Z‐scheme photocatalytic systems are also presented.

## 1 Introduction

**Figure 1:**
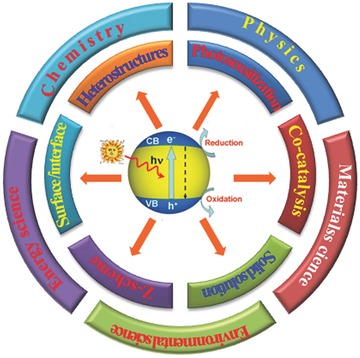
Schematic illustration of research field of photocatalysts.

Energy and environmental issues are important topics on a global level. To tackle the issue of the depletion of fossil fuels and their environmental misdeeds, the exploration of renewable and clean energy resources and the development of eco‐friendly practical systems for environmental remediation have been drawing increasing attention (**Figure** **1**). Semiconductor photocatalyst systems can be used to split water into H_2_ and O_2_, to photo‐reduce CO_2_ into renewable fuels such as CH_3_OH, CH_4_, and CO, and to decompose various organic contaminations to remedy the environment. These methods use solar energy as the source of photons to promote reactions.^1^, ^2^ Photocatalytic reaction processes primarily involve three main steps: (1) the photogeneration of electron–hole pairs through the absorption of solar light of the energy larger than the band gap of photocatalyst, (2) the charge separation and migration onto the surface without recombination, and (3) the reduction/oxidation reaction on the surface of photocatalyst.^3^, ^4^, ^5^ The efficiency of a photocatalyst is primarily determined by the balance of the thermodynamics and kinetics of these processes. It is difficult for a single‐component photocatalyst to simultaneously possess wide light‐absorption range and strong redox ability, because wide light‐absorption range that needs to narrow the semiconductor bandgap is incompatible with strong redox ability that should widen the semiconductor bandgap. This is reason that a more negative potential of the conduction band (CB) and a more positive potential of the valence band (VB) are beneficial for the reduction and oxidation of reactants, respectively. Although some metal oxides (e.g., TiO_2_ and WO_3_) and non‐oxides (e.g., CdS and Ta_3_N_5_) are widely used as photocatalysts, their performance of the photocatalytic reaction is still very low.^2^, ^6^ The construction of heterostructured photocatalyst systems (usually type‐II heterojunctions) that comprise multiple components or multiple phase is one effective strategy to improve photocatalytic efficiency because of the tunable band structures and efficient electron‐hole separation and transportation.^3^ However, the redox ability of photoexcited electrons and holes on reaction sites are weakened, leading to lower redox ability.^7^, ^8^ Therefore, it is necessary to develop a novel photocatalytic system to overcome the aforementioned problems and significantly improve the efficiency of the photocatalytic reaction.

**Figure 2:**
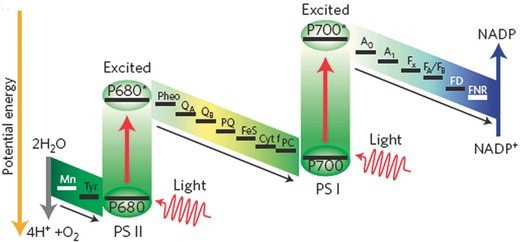
Charge separation mechanism in natural photosynthesis.^8^ Reproduced with permission.^8^ Copyright 2012, Nature Publishing Group.

**Figure 3:**
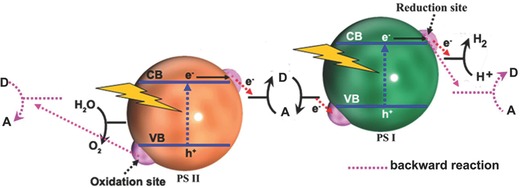
Forward and backward reactions in a Z‐scheme system with shuttle redox mediators.^18^ Reproduced with permission.^18^ Copyright 2005, American Chemical Society.

In nature, H_2_O and CO_2_ are converted into O_2_ and carbohydrate through natural photosynthesis (NPS) in green plants, as shown in **Figure** **2**. This photosystem, which looks like English letter “Z”, known as the Z‐scheme, involves a two‐step photoexcitation.^8^, ^9^, ^10^ Photosystem I (PS I) and photosystem II (PS II) harvest solar energy and pump electrons to a higher electronic state (excitation), which are connected in series with an electron transfer chain (electron mediator). The electrons in PS II flowed from the electron transport chain leads to the reduction of co‐enzyme NADP^+^ into NADP that is used to fix CO_2_ into carbohydrate in the dark reaction, and the water oxidation occurs at a manganese calcium oxide cluster in PS II.^8^, ^9^, ^10^, ^11^ The efficiency of charge separation in the reaction center of PS I protein is close to 100%. The artificial Z‐Scheme system that mimics natural photosynthesis of green plant consists of two connected semiconductor photocatalysts, which has the merit of keeping electrons/holes with stronger reduction/oxidation abilities on different active sites. In a typical Z‐scheme photocatalytic system, two different photocatalysts are combined using an appropriate shuttle electron mediator, as shown in **Figure** **3**.^18^ Visible light can be utilized more efficiently because the range of solar energy for driving each photocatalyst is reduced, and electrons/holes with stronger reduction/oxidation abilities in PS I and PS II could be produced.^12^, ^13^

Z‐scheme water splitting was first reported by Bard et al. in 1979.^14^ Since then, Z‐scheme water splitting has attracted considerable attention. Reversible redox mediators (e.g., Fe^3+^/Fe^2+^, IO_3_^–^/I^–^, NO_3_^–^/NO_2_^–^) are usually served as electron transport chain in Z‐scheme water splitting.^4^ However, backward reactions easily happen in the reversible redox mediator Z‐scheme photocatalytic system, which are thermodynamically downhill in most cases. Redox mediators could cause undesirable backward reactions involving redox mediators, such as competitive oxidation of I^–^ by holes in the O_2_ evolution photocatalyst when using IO_3_^–^/I^–^ as redox mediator. Therefore, the reaction efficiency of reversible redox mediator Z‐scheme photocatalytic system could be destroyed. In addition, the redox mediators also strongly absorb the visible light, reducing the light absorption of semiconductor photocatalysts. Thus, the Z‐scheme photocatalytic systems without reversible redox pair mediators or with solid state electron mediators are necessary to be developed for water splitting.^3^, ^4^, ^7^ The noble‐metal particles (such as Au, Ag) and graphene were explored as an solid electron mediator for the Z‐scheme photocatalytic system. Thus, the aforementioned backward reactions can be perfectly avoided. Nevertheless, the noble‐metal particles (such as Au, Ag) still strongly absorb part of the visible light due to surface plasmon resonance (SPR) effects, also reducing light absorption of semiconductor photocatalysts. So direct Z‐scheme systems without the reversible redox pair mediators were also exploited in recent years.^27^, ^33^ Several reviews related to Z‐scheme water splitting have been reported,^4^, ^8^, ^12^ and all‐solid‐state Z‐scheme photocatalytic systems without redox pair were especially summarized, which mainly exhibit the obvious differences of all‐solid‐state Z‐scheme systems in the preparation methodology, working mechanism, properties and application.^7^ Some Z‐scheme examples were also partly presented in our previous reviews about heterostructured photocatalysts^3^ and CO_2_ photoreduction.^15^

Based on the discussion above, we know that the connection between two photocatalysts is crucial for constructing efficient Z‐scheme photocatalytic systems. Many works studied different connection modes of Z‐scheme photocatalytic systems.^13^, ^17^, ^18^, ^19^, ^20^, ^22^, ^23^, ^24^, ^25^, ^26^, ^27^, ^28^, ^29^, ^30^, ^31^, ^32^, ^33^, ^34^, ^35^, ^36^, ^37^, ^38^, ^39^, ^40^, ^41^, ^43^, ^44^, ^45^, ^46^, ^47^, ^48^, ^49^, ^50^, ^51^, ^52^, ^53^, ^54^, ^55^, ^56^, ^57^, ^58^, ^59^, ^60^, ^61^, ^62^, ^63^, ^64^, ^65^, ^66^, ^67^, ^68^, ^69^, ^70^, ^71^, ^72^, ^73^, ^74^, ^75^, ^76^, ^77^, ^78^, ^79^, ^80^, ^81^, ^82^, ^83^, ^84^, ^85^, ^86^, ^87^, ^88^, ^89^, ^90^, ^91^, ^92^, ^93^, ^94^, ^95^, ^96^, ^97^ The present review concisely summarizes and highlights recent state‐of‐the‐art accomplishments of Z‐scheme systems, which was categorized through diverse modes, including i) with shuttle redox mediators, ii) without electron mediators, and iii) with solid‐state electron mediators. The challenges and prospects for future development of Z‐scheme photocatalytic systems are also presented.

## 2 Z‐Scheme Systems with Shuttle Redox Mediators

As shown in Figure 3, this kind of Z‐scheme photocatalytic system consists of two different photocatalysts and an acceptor/donor (A/D) pair (so‐called shuttle redox mediator).^4^, ^8^, ^16^, ^17^, ^18^ No physical contact exists between PS I and PS II. Under solar light radicalization, the forward reactions on a H_2_ evolution (PS I) photocatalyst occur as follows:

$$2H^{+}+2e^{-}\to H_{2}\left(\mathrm{photoreduction}\;\;\mathrm{of}\;H^{+}\;\mathrm{to}\;H_{2}\right)$$

$$D+nh^{+}\to A\left(\mathrm{photooxidation}\;\mathrm{of}\;D\;\;\mathrm{to}\;A\right)$$

The forward reactions on an O_2_ evolution (PS II) photocatalyst should occur as follows:

$$A+ne^{-}\to D\left(\mathrm{CB}\;\mathrm{of}\;\mathrm{PS}\;\mathrm{II}\right)$$

$$2H_{2}O+4h^{+}\to O_{2}+4H^{+}\left(\mathrm{VB}\;\;\mathrm{of}\;\;\mathrm{PS}\;\;\mathrm{II}\right)$$

However, the demonstration of the simultaneous evolution of H_2_ and O_2_ is extremely difficult in a Z‐scheme system because the backward reactions also easily proceed over each photocatalyst,^7^, ^17^ as follows:

$$A+ne^{-}\to D\left(\mathrm{CB}\;\mathrm{of}\;\mathrm{PS}\;I\right)$$

$$D+nh^{+}\to A\left(\mathrm{VB}\;\mathrm{of}\;\mathrm{PS}\;\mathrm{II}\right)$$

The electron acceptor (A) and donor (D) react with the photogenerated electrons in the CB of PS I and holes in the VB of PS II, respectively, resulting in the obviously decrease in the effective number of photogenerated electrons and holes. Thus, it is critical to suppress the backward reactions involving redox mediators that are thermodynamically more favorable than water splitting. In 2010, Domen et al. succeeded in water splitting using a Z‐scheme system consisting of Pt‐loaded ZrO_2_/TaON and Pt‐loaded WO_3_ as the H_2_‐ and O_2_‐evolution photocatalysts, respectively, in the presence of an IO_3_^–^/I^–^ redox mediator.^19^ The undesirable backward reactions, such as oxidation of I^–^ ions on the Pt‐loaded WO_3_ and the reduction of IO_3_^–^ on the Pt‐loaded ZrO_2_/TaON, were significantly minimized. As a result, the highest apparent quantum efficiency (AQE) of 6.3% at 420 nm was achieved. This high selectivity for the forward reactions in the Z‐scheme system was also demonstrated by using Pt‐SrTiO_3_:Cr/Ta, Pt‐WO_3_, and the IO_3_^–^/I^–^ redox mediator.^17^ Additionally, the forward reactions in the Z‐scheme system were promoted by the presence of a nanoparticulate cocatalyst (e.g., Pt, Rh, NiO*_x_*, RuO_2_), that was loaded onto the photocatalyst surface to produce active sites and reduce the activation energy for the surface chemical reactions.^4^, ^12^ The behavior of different cocatalysts (e.g., Pt, Rh, Ni, Ru, Fe_2_O_3_, and RuO_2_) loaded on SrTiO_3_:Rh/WO_3_ visible‐light‐driven Z‐scheme photocatalysis systems for H_2_ and O_2_ evolution with Fe^3+^/Fe^2+^ as the electron mediator were investigated, and the water splitting was obviously enhanced in comparison with the non‐loaded system.^20^ The water splitting of the system using the Pt cocatalyst decreased as the partial pressures of the evolved H_2_ and O_2_ were increased. However, such deactivation was not observed for the system using the Ru cocatalyst due to the significant suppression of the backward reactions. Moreover, the selective catalysis is achieved via the introduction of a proper cocatalyst on the photocatalyst surface. Mn_3_O_4_, RuO_2_, IrO_2_, and CoO*_x_* nanoparticles were found to function as O_2_ evolution cocatalysts.^21^ For instance, RuO_2_‐loaded TaON is an effective photocatalyst for O_2_ evolution to achieve water splitting in combination with Pt‐loaded TaON as a H_2_ evolution photocatalyst with an IO_3_^–^/I^–^ redox mediator.^22^ TaON alone was not applicable for the O_2_ evolution due to the efficiently competitive oxidation of I^–^. However, loading RuO_2_ on the TaON surface enables water oxidation even in the presence of I^–^.^4^, ^22^, ^23^ IrO_2_‐loaded Ta_3_N_5_ or Pt‐BaTaO_2_N is another interesting example that allows water oxidation in the presence of IO_3_^–^/I^–^, respectively.^24^, ^25^ The choice of the reaction pH plays a key role in effectively improving the efficiency of the Z‐scheme system.^4^, ^17^, ^18^ A pH‐dependent Z‐scheme system based on Pt/anatase TiO_2_ and rutile TiO_2_ as a H_2_ and O_2_ evolution photocatalyst, respectively, achieved much higher evolution rates of H_2_ and O_2_ at pH > 9.^17^

All primary semiconductor‐based Z‐scheme systems with shuttle redox mediators are summarized in **Table** **1**. Nevertheless, these Z‐scheme systems have various negative effects, such as back reactions for the water splitting reaction. The redox mediators may also strongly absorb visible light, reducing the light absorption of the semiconductor photocatalysts. Additionally, it is often difficult to maintain long‐term stability and active state for the redox mediators, resulting in a decrease in reaction rates. To avoid these disadvantages, Z‐scheme systems without redox mediators have attracted much attention because they eliminate these limitations.^7^, ^26^

**Table 1:** Z‐Scheme photocatalyst system with shuttle redox mediators

PS I (available wavelength [nm])	PS II (available wavelength [nm])	Light source	Redox mediators	Application	Activity [μmol h^–1^]		Efficiencya)	Ref. (year)
Fe^2+^ (<280)	RuO_2_/WO_3_ (<460)	Hg lamp (400 W)	Fe^2+^/Fe^3+^	water splitting	H_2_:38	O_2_:15		16 (1997)
Pt/SrTiO_3_:Cr–Ta (<700)	Pt/WO_3_ (<460)	Xe lamp (300 W)	I^–^/IO_3_^–^	water splitting	H_2_:0.21	O_2_:0.11	AQE = 0.1% (420 nm)	64 (2001)
Pt/TiO_2_‐anatase (<400)	TiO_2_‐rutile (<400)	Hg lamp (400 W)	I^–^/IO_3_^–^	water splitting	H_2_:180	O_2_:90		65 (2001)
Pt/SrTiO_3_ (<520)	BiVO_4_ (<520)	Xe lamp (300 W)	Fe^2+^/Fe^3+^	water splitting	H_2_:15	O_2_:7.2	AQE = 0.3% (440 nm)	66 (2004)
Pt/TaON (<500)	Pt/WO_3_ (<460)	Xe lamp (300 W)	I^–^/IO_3_^–^	water splitting	H_2_:24	O_2_:12	AQE = 0.4 % (420 nm)	67 (2005)
m‐ZrO_2_/TaON (<500)	Pt/WO_3_ (<460)	Xe lamp (300 W)	I^–^/IO_3_^–^	water splitting	H_2_:4.1	O_2_:2		68 (2008)
Pt/ATaO_2_N (A = Ca, Sr, Ba) (<500)	Pt/WO_3_ (<460)	Xe lamp (300 W)	I^–^/IO_3_^–^	water splitting	H_2_:6.6	O_2_:3.3	AQE = 0.1% (420–440 nm)	69 (2008)
Ru/SrTiO_3_:Rh (<520)	BiVO_4_ (<520)	Xe lamp (300 W)	Fe^2+^/Fe^3+^	water splitting	H_2_:18.9	O_2_:8.9	AQE = 0.3% (420 nm)	20 (2008)
Pt/H_2_K_2_Nb_6_O_17_ (<750)	IrO_2_‐Pt/WO_3_ (<460)	Xe lamp (300 W)	I_3_^−^/I^−^	water splitting	H_2_:2.1	O_2_:1.0		70 (2009)
Pt/SrTiO_3_ (<520)	BiVO_4_ (<520)	Xe lamp (300 W)	Fe^2+^/Fe^3+^	water splitting	H_2_:40	O_2_:19	AQE = 1.7% (420 nm)	31 (2009)
Pt/ZrO_2_/TaON (<520)	Pt/WO_3_ (<460)	Xe lamp (300 W)	I^–^/IO_3_^–^	water splitting	H_2_:7.5	O_2_:3.8	AQE = 6.3%	19 (2010)
Pt/ZrO_2_/TaON (<520)	Ir/R‐TiO_2_/Ta_3_N_5_ (<600)	Xe lamp (300 W)	I^–^/IO_3_^–^	water splitting	H_2_:7.0	O_2_:1.0		24 (2010)
Pt/ZrO_2_/TaON (<520)	RuO_2_/TaON(<520)	Xe lamp (300 W)	I^–^/IO_3_^–^	water splitting	H_2_:8.0	O_2_:3.0		23 (2011)
Pt/SrTiO_3_:Rh (<520)	Ru/SrTiO_3_:In/V(<520)	Xe lamp (300 W)	I^–^/IO_3_^–^	water splitting			AQE = 0.33% (360 nm)	71 (2012)
Pt/SrTiO_3_:Cr/Ta (<550)	Cs^+^−PtO*_x_*/WO_3_ (<460)	Xe lamp (300 W)	I^–^/IO_3_^–^,I^–^/I_3_^–^	water splitting	H_2_:40	O_2_:20	AQE = 1.5% (420 nm)	72 (2013)
Ru/SrTiO_3_:Rh (<520)	BiVO_4_ (<520)	Xe lamp (300 W)	[Co(bpy)_3_]^3+/2+^ and [Co(phen)_3_]^3+/2+^	water splitting	H_2_:94	O_2_:38	AQE = 2.1% (420 nm)	73 (2013)
Ru/SrTiO_3_:Rh (<520)	BiVO_4_ (<520)	Xe lamp (300 W)	Fe^2+^/Fe^3+^	water splitting	H_2_:1.2	O_2_:0.6	AQE = 4.2 % (420 nm)	74 (2013)
BaZrO_3_–BaTaO_2_N(<690)	PtO*_x_*/WO_3_ (<460)	Xe lamp (300 W)	I^–^/IO_3_^–^	water splitting	H_2_:150	O_2_:62		75 (2013)
Pt/Sm_2_Ti_2_S_2_O_5_ (<650)	TiO_2_‐rutile (<387)	Hg lamp (450 W)	I^–^/IO_3_^–^	water splitting	H_2_:45	O_2_:16		76 (2014)
Pt/g‐C_3_N_4_ (<450)	Pt/WO_3_(<460)	Xe lamp (300 W)	I^–^/IO_3_^–^	water splitting	H_2_:74	O_2_:37		77 (2014)
Pt/carbon nanodots	WO_3_(<460)	Xe lamp (300 W)	I^–^/IO_3_^–^	H_2_‐evolution	H_2_:1330			78 (2015)
Pt/MgTa_2_O_6–y_N*_x_*/TaON(<570)	PtO*_x_*‐WO_3_(<460)	Xe lamp (300 W)	I^–^/IO_3_^–^	water splitting	H_2_:108.3	O_2_:55.3	AQE = 6.8% (420 nm)	79 (2015)

^a)^AQE = apparent quantum efficiency.

## 3 Z‐Scheme Systems Without Redox Mediators

**Figure 4:**
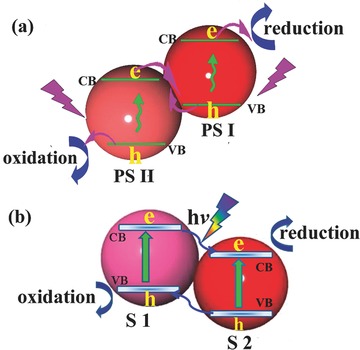
Schematic illustrations of (a) direct Z‐scheme system and (b) type‐II heterojunction.^3^ Reproduced with permission.^3^

A direct Z‐scheme system without redox mediators was schematically illustrated in **Figure** **4**a. Under solar light irradiation, the photoexcited electrons in PS II with a relatively low CB may recombine with the holes in PS I that have a relatively high VB at the solid heterostructure interface. Then, more oxidative holes and reductive electrons can be retained on different counterparts, resulting in an enhancement in the photocatalytic efficiency. Notably, the backward reactions in a Z‐scheme system with redox mediators are primarily suppressed because of the absence of redox mediators. Furthermore, the shielding effect of the irradiated incident light that is caused by the redox mediators can also be significantly eliminated.

**Figure 5:**
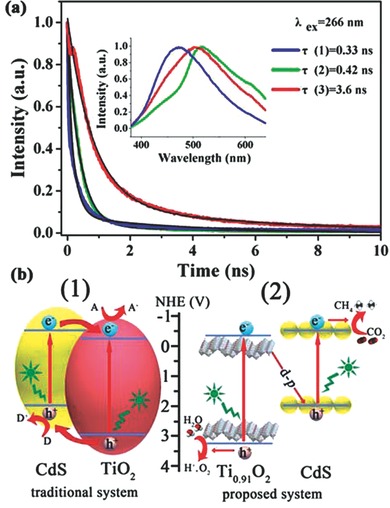
a) PL decay traces of Ti_0.91_O_2_ hollow spheres (blue), CdS hollow spheres (green) and Ti_0.91_O_2_/CdS hollow spheres (red). The inset is the PL emission spectra of Ti_0.91_O_2_ hollow spheres (blue) and Ti_0.91_O_2_/CdS hollow spheres (red). b) Schematic illustration of a traditional TiO_2_‐CdS system (route 1) and an artificial Z‐scheme system (route 2).^27^ Reproduced with permission.^27^ Copyright 2015, The Royal Society of Chemistry.

It is necessary to investigate the occurrence of the direct Z‐scheme system instead of the type‐II heterojunction mechanism (as shown in Figure 4b) when two semiconductors contact without redox mediators.^27^ The type‐II heterojunction facilitated the transfer of electrons from CB of semiconductor 1 (S 1) to that of semiconductor 2 (S 2) and the opposite transfer route of the holes. The different electron‐hole transfer mechanisms between direct Z‐scheme photocatalytic system and type‐II heterojunction system could be investigated by photoluminescence (PL) spectra and transient time‐resolved PL decay measurements. For instance, our group constructed ultrathin multilayer hollow spheres that consist of alternating Ti_0.91_O_2_ nanosheets (≈0.75 nm) and CdS nanoparticles (≈5–6 nm) via an exquisite layer‐by‐layer self‐assembly to realize a redox mediator‐free direct Z‐scheme system for the photocatalytic reduction of CO_2_ into CH_4_ (**Figure** **5**). The indirect optical transition effect in the multilayer Ti_0.91_O_2_/CdS hollow spheres was confirmed via PL spectra and transient time‐resolved PL decay measurements. The results demonstrate successful construction of an artificial Z‐scheme system, in which excited electrons in the CB of Ti_0.91_O_2_nanosheets recombined with holes in the VB of CdS NPs via d–p conjugation. This system was completely different from the traditional type‐II TiO_2_–CdS heterostructure system.^27^ Our research may provide a new viewpoint for the tailoring and constructing of a hybrid nanostructure of semiconductors for photocatalysis.

The contact interface of two different photocatalysts has a significant impact on the charge transfer in a direct Z‐scheme system. The physical contact formation between the PS I and PS II is one of contact modes in direct Z‐scheme system, which is usually based on the electrostatic adsorption due to opposite charges.^28^, ^29^ In a direct Z‐scheme system of WO_3_/CaFe_2_O_4_, the CaFe_2_O_4_ surface was negatively charged and efficiently adsorbed cation species in aqueous solution at pH = 7.^30^ A mild neutral condition was effective for promoting the selective nucleation of WO_3_ particles on the surface of the CaFe_2_O_4_, resulting in a significant increase in the photocatalytic reaction rate of the WO_3_/CaFe_2_O_4_ composite for the decomposition of gaseous acetaldehyde under visible‐light irradiation. Similarly, in a Z‐scheme BiVO_4_‐Ru/SrTiO_3_:Rh system, the highest photocatalytic activity was achieved at a pH of 3.5 because BiVO_4_‐Ru and SrTiO_3_:Rh have negative and positive charges, respectively.^31^ A favorable solid–solid contact interface was formed between BiVO_4_‐Ru and SrTiO_3_:Rh, resulting in a high AQE of 1.7% at 420 nm. Therefore, adjustment of the pH should be considered during the formation of a solid‐solid contact interface between PS I and PS II. Furthermore, mechanical force is another method to form a solid–solid contact interface. A highly active Z‐scheme NaNbO_3_/WO_3_ photocatalyst was prepared using a facile ball milling method.^32^ The photocatalytic activity of NaNbO_3_/WO_3_ was higher than individual NaNbO_3_ and WO_3_ due to the fast recombination between the photogenerated holes of NaNbO_3_ and the photogenerated electrons of WO_3_.

The chemical contact is the other contact mode to form intimate interface between PS I and PS II for effective carriers transfer, due to the surface conjugation via chemical bond. Wet chemistry is widely used to synthesize direct Z‐scheme systems.^28^, ^33^, ^34^ A Na_2_S solution was added dropwise into a suspension of CdCl_2_ and Zn(OH)_2_ precipitate, resulting in a Z‐scheme ZnO/CdS photocatalytic system.^33^, ^34^ ZnO/CdS are highly active photocatalysts for H_2_ evolution under simulated solar light irradiation, due to the strong phase interaction by the binding energy shift of Zn 2p_3/2_, Cd 3d_5/2_, O 1s, and S 2p in the ZnO/CdS heterostructure in contrast to the pure ZnO and CdS. Z‐scheme BiVO_4_/g‐C_3_N_4_ composites that the facet coupling occurred between the g‐C_3_N_4_ (002) and BiVO_4_ (121) were obtained by calcination of a mixture of appropriate amounts of BiVO_4_ and g‐C_3_N_4_ at 400°C for 4 h.^35^ Similarly, other Z‐scheme composites, such as MoO_3_/g‐C_3_N_4_, ZnO/g‐C_3_N_4_, and WO_3_/g‐C_3_N_4_, have also been synthesized through calcination process.^36^, ^37^, ^38^ Additionally, hydrothermal reactions were extensively applied to the formation of direct Z‐scheme systems.^35^, ^39^, ^40^ Direct Z‐scheme Si/TiO_2_ composites were fabricated via a facile hydrothermal reaction with tetrabutyltitanate and as‐prepared Si nanospheres.^39^

The direct Z‐scheme system can work in both liquid‐phase and gas‐phase environments, which are widely used to split water, photoreduce CO_2_, and decompose various organic contaminations (see **Table** **2**). Solar‐driven water splitting has been achieved under simulated sunlight using direct Z‐scheme Si/TiO_2_ nanotree‐structures.^41^ The photogenerated electrons in Si and the holes in TiO_2_ moved to the surface to perform water splitting as the photogenerated holes in Si and the electrons in TiO_2_ recombined at the Ohmic contact between the Si and TiO_2_. The solar‐to‐fuel conversion efficiency was approximately 0.12%, which is comparable to that of natural photosynthesis. Additionally, the Z‐scheme Si/TiO_2_ consisted of porous Si nanospheres and TiO_2_ nanosheets that achieved high activity towards CO_2_ reduction into methanol with a maximum photonic efficiency of 18.1%.^39^ The direct Z‐scheme NaNbO_3_/WO_3_ photocatalyst exhibited a relatively much higher photocatalytic activity, and the rate constants of RhB and MB degradation for NaNbO_3_/WO_3_ were 4.9 times and 3.4 times than that of pure WO_3_, respectively. This attributed to the fast recombination between the photogenerated holes of NaNbO_3_ and the photogenerated electrons of WO_3_.^32^ As a promising photocatalyst, graphite‐like carbon nitride (g‐C_3_N_4_), which consists of only carbon and nitrogen, is a sustainable, cost‐effective and environmental‐friendly semiconductor that has attracted extensive interest.^42^ The combination of g‐C_3_N_4_ with other appropriate semiconductors for construction of a direct Z‐scheme system can effectively improve the photocatalytic performance.^35^, ^36^, ^37^, ^38^, ^40^, ^43^, ^44^, ^45^ Recently, a direct Z‐schemeg‐C_3_N_4_/AgBr photocatalyst was prepared by loading AgBr nanoparticles on a protonated g‐C_3_N_4_ matrix.^43^ Fast recombination may occur between the photogenerated electrons in the CB of AgBr and the photogenerated holes in the VB of g‐C_3_N_4_. As a result, the photodegradation rate of methyl orange (MO) over the g‐C_3_N_4_/AgBr photocatalyst was 33.8 and 2.1 times higher than that over pure g‐C_3_N_4_ and AgBr samples, respectively.

**Table 2:** Direct Z‐Scheme systems

PS I (available wavelength [nm])	PS II (available wavelength [nm])	Light source	Application	Activity	Efficiencya)	Ref. (year)
CaFe_2_O_4_ (<670)	WO_3_ (<460)	Xe lamp (150 W)	photodegradation	CH_3_CHO degradation		30 (2013)
NaNbO_3_	WO_3_ (<460)	Hg lamp (375 W)	photodegradation	RhB degradation	DE = 96% (80 min)	32 (2013)
g‐C_3_N_4_ (<450)	TiO_2_ (<387)	UV lamp (15 W)	photodegradation	HCHO degradation	DE = 94% (1 h)	80 (2013)
Si (<1107)	TiO_2_ (<387)	Xe lamp (300 W)	photodegradation	RhB degradation	DE = 99% (1 h)	39 (2014)
g‐C_3_N_4_ (<450)	ZnO:N (395)	Xe lamp (300 W)	photodegradation	RhB degradation	DE = 99% (1 h)	37 (2014)
g‐C_3_N_4_ (<450)	SrTiO_3_:N (<420)	Xe lamp (300 W)	photodegradation	RhB degradation	DE = 98% (1 h)	44 (2014)
g‐C_3_N_4_ (<450)	MoO_3_ (<450)	Xe lamp (350 W)	photodegradation	MO degradation	DE = 91% (2 h)	36 (2014)
g‐C_3_N_4_ (<450)	BiOCl (<375)	Xe lamp (300 W)	photodegradation	RhB degradation	DE = 99% (1 h)	81 (2014)
AgI (<521)	β‐Bi_2_O_3_ (<443)	UV‐LED (3W)	photodegradation	MO degradation	DE = 99% (4 h)	82 (2015)
Bi_20_TiO_32_ (<550)	g‐C_3_N_4_ (<450)	Xe lamp (300W)	photodegradation	RhB degradation	DE = 99% (20 min)	83 (2015)
g‐C_3_N_4_ (<450)	BiVO_4_ (<520)	Xe lamp (500W)	photodegradation	RhB degradation	DE = 85% (5 h)	35 (2015)
g‐C_3_N_4_ (<450)	AgBr (<490)	Xe lamp (300W)	photodegradation	MO degradation	DE = 78.9% (2 h)	43 (2015)
CuO (<730)	TiO_2_ (<387)	Hg lamp (250W)	CO_2_ Reduction	methyl:1600 μmol g^–1^h^–1^		84 (2011)
CdS (<540)	TiO_2_ (<387)	Hg lamp (250W)	CO_2_ Reduction	C_2_H_12_O_2_: 22.21.57 μmol g^–1^h^–1^		34 (2014)
				C_6_H_10_O: 20 μmol g^–1^h^–1^		
Si (<1107)	TiO_2_ (<387)	Xe lamp (300W)	CO_2_ Reduction	CH_3_OH	PE = 18.1%	39 (2014)
Si (<1107)	TiO_2_ (<387)	Xe lamp (300W)	CO_2_ Reduction	CH_4_:0.14 μmol g^–1^h^–1^		39 (2014)
Bi_2_WO_6_ (<470)	g‐C_3_N_4_ (<450)	Xe lamp (300W)	CO_2_ Reduction	CO:5.19 μmol g^–1^h^–1^		40 (2015)
CdS (<500)	Ti_0.91_O_2_ (<326)	Xe lamp (300W)	CO_2_ Reduction	CH_4_:0.1 μmol g^–1^h^–1^		27 (2015)
				O_2_:0.18 μmol g^–1^h^–1^		
CdS (<540)	ZnO (<382)	Xe lamp (300W)	water splitting	H_2_:3870 μmol g^–1^h^–1^		33 (2009)
Si (<1060)	TiO_2_ (<387)	Xe lamp (300W)	water splitting	H_2_:875 μmol g^–1^h^–1^	CE = 0.12%	41 (2013)
				O_2_:458 μmol g^–1^h^–1^		
Ru/SrTiO_3_:Rh (<520)	Ir/CoO*_x_*Ta_3_N_5_ (<600)	Xe lamp (300W)	water splitting	H_2_:23 μmol g^–1^h^–1^		85 (2013)
				O_2_:12 μmol g^–1^h^–1^		
g‐C_3_N_4_ (<450)	WO_3_ (<460)	Xe lamp (300W)	water splitting	H_2_:110 μmol g^–1^h^–1^	AQE = 0.90% (405 nm)	38 (2014)

^a)^PE = photonic efficiency; CE = conversion efficiency; AQE = apparent quantum efficiency.

Although direct Z‐scheme systems effectively improve photocatalytic activities due to the recombination of photoexcited electrons from the CB of PS II and holes from the VB of PS I at the interface, the photoexcited carrier transport process at the interface between PS II and PS I is often poor due to surface relaxation and the recombination of charge carriers within each component. Therefore, the design of a new structural system with pathways for quick transport of charge carriers at the interface is highly desired.

## 4 Z‐Scheme Systems with Solid State Electron Mediators

**Figure 6:**
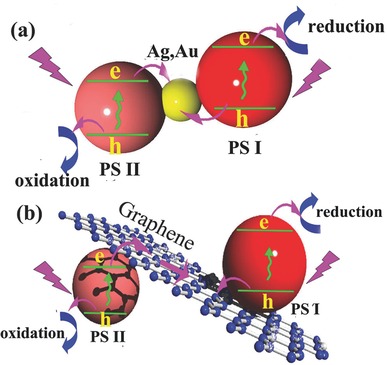
a) Schematic illustrations of a Z‐scheme in the presence of Au with Ag as the electron‐mediator. b) Schematic illustrations of a Z‐scheme in the presence of RGO as the electron‐mediator.^3^ Reproduced with permission.^3^

As shown in **Figure** **6**, the Z‐scheme photocatalytic system consists of two different photocatalysts (PS I and PS II) and a solid electron mediator at the interface of two semiconductors, and their photocatalytic applications were listed in **Table** **3**. Noble‐metal particles (such as Au, Ag) and reduced graphene oxide (RGO) were explored as electron mediators for the Z‐scheme system, and a high efficiency of the charge‐carrier separation and transport can be achieved at the interface of the two semiconductors.^26^, ^47^, ^48^

**Table 3:** Z‐scheme with solid state electron mediators

PS I (available wavelength [nm])	PS II (available wavelength [nm])	Light source	Electron mediators	Application	Activitya)	Efficiencyb)	Ref. (year)
AgBr (<490)	Bi_2_WO_6_ (<470)	Xe lamp (300 W)	Ag	photodegradation	Procion red degradation	DE = 85% (1 h)	86 (2009)
CdS (<540)	TiO_2_ (<387)	Hg lamp (20 W)	Au	photodegradation	MB degradation	DE = 72% (2 h)	87 (2009)
CaFe_2_O_4_ (<670)	WO_3_ *λ* < 460	Xe lamp (150 W)	Ag	photodegradation	CH_3_CHO degradation	DE = 99% (48 h)	63 (2009)
AgCl (<382)	H_2_WO_4_·H_2_O (<500)	fluorescence lamp (55 W)	Ag	photodegradation	MO degradation	DE = 87% (4 h)	54 (2011)
AgBr (<490)	BiOBr (<427)	Xe lamp (500 W)	Ag	photodegradation	RhB degradation	DE = 99% (44 min)	57 (2012)
AgI (<521)	AgBr (<490)	Xe lamp (500 W)	Ag	photodegradation	MO degradation	DE = 97% (16 min)	88 (2012)
AgCl (<382)	TaON (<453)	Xe lamp (300 W)	Ag	photodegradation	RhB degradation	DE = 96.6% (140 min)	89 (2013)
					AO7 degradation	DE = 98% (100 min)	
AgI (<521)	Ag_3_PO_4_ (<512)	Xe lamp (500 W)	Ag	photodegradation	MO degradation	DE = 84% (18 min)	58 (2013)
AgCl (<382)	Bi_20_TiO_32_ (<540)	Xe lamp (300 W)	Ag	photodegradation	RhB degradation	DE = 82% (5 min)	55 (2013)
g‐C_3_N_4_ (<450)	Ag_3_PO_4_ (<512)	Xe lamp (300 W)	Ag	photodegradation	MO degradation	DE = 99% (5 min)	45 (2014)
RGO (<885)	AgCl (<382)	LED lamp	Ag	photodegradation	MB degradation	DE = 99% (60 min)	90 (2014)
AgCl (<382)	α/β‐Bi_2_O_3_ (<443)	Xe lamp (300 W)	Ag	photodegradation	RhB degradation	DE = 98% (30 min)	53 (2014)
					AO7 degradation	DE = 99% (30 min)	
\\Cu_2_O (<450)	Na*_x_*H_2−_ *_x_*Ti_3_O_7_ (<345)	Xe lamp (500 W)	Au	photodegradation	RhB degradation	DE = 80% (1 h)	91 (2015)
AgBr (<490)	Ag_2_CO_3_ (<480)	Xe lamp (500 W)	Ag	photodegradation	RhB degradation	DE = 99% (30 min)	52 (2015)
					MO degradation	DE = 99% (30 min)	
SiC (<480)	Ag_3_PO_4_ (<512)	Xe lamp (300 W)	Ag	photodegradation	MO degradation	DE = 97% (15 min)	50 (2015)
InP/[MCE_2_‐A + MCE_4_] (<918)	Pt/TiO_2_ (<387)	Xe lamp (300 W)	Cu	CO_2_ reduction	HCOOH: 0.22 μmol cm ^2^ h^–1^		92 (2011)
InP/[RuCP] (<900)	Reduced SrTiO_3_ (<400)	Solar simulator (AM1.5)	Ag	CO_2_ reduction	HCOOH: 0.48 μmol h^–1^	CE = 0.14%	93 (2013)
Pt/Fe_2_V_4_O_13_ (<677)	CdS (<540)	Xe lamp (300 W)	RGO	CO_2_ reduction	CH_4_: 1.57 μmol g^–1^h^–1^		48 (2015)
g‐C_3_N_4_ (<450)	Ag_3_PO_4_ (<512)	Xe lamp (500 W)	Ag	CO_2_ reduction	C_2_H_5_OH:57.5 μmol g^–1^h^–1^		94 (2015)
PbBi_2_Nb_1.9_Ti_0.109_<430	WO_3_<460	Xe lamp (450 W)	W	water splitting	H_2_: 49.3 μmol g^–1^h^–1^		62 (2006)
					O_2_: 741 μmol g^–1^h^–1^		
Pt/CdS<540	TiO_2_<400	Xe lamp (500 W)	Au	water splitting	H_2_: 10 μmol g^–1^h^–1^		26 (2006)
Pt/CdS (<540)	TiO_1.96_C_0.04_ (<477)	Xe lamp (300 W)	Au	water splitting	H_2_: 433.2 μmol g^–1^h^–1^		47 (2011)
					H_2_: 11 μmol g^–1^h^–1^		
Ru/SrTiO_3_:Rh (<520)	BiVO_4_ (<520)	Xe lamp (300 W)	RGO	water splitting	O_2_: 5.5 μmol g^–1^h^–1^		61 (2011)
CdS (<540)	ZnO (<387)	Xe lamp (300 W)	Cd	water splitting	H_2_: 1920 μmol g^–1^h^–1^		95 (2012)
CdS (<540)	TiO_2_ (<387)	Xe lamp (750 W)	Au	water splitting	H_2_: 64 μmol g^–1^h^–1^		96 (2013)
ZnRh_2_O_4_ (<1033)	Ag_1–_ *_x_*SbO_3–_ *_y_* (<460)	Xe lamp (300 W)	Ag	water splitting	H_2_: 0.0168 μmol g^–1^h^–1^	AQE = 0.090% (365 nm)	51 (2014)
					O_2_: 0.0084 μmol g^–1^h^–1^		
Ru/SrTiO_3_:La/Rh (<500)	Ir/CoO*_x_*/Ta_3_N_5_ (<600)	Xe lamp (300 W)	Ir	water splitting	H_2_: 280 μmol g^–1^h^–1^	AQE = 1.1% (420 nm)	97 (2014)
					O_2_: 140 μmol g^–1^h^–1^		
CuGaS_2_ (<520)	TiO_2_ (<387)	Xe lamp (300 W)	RGO	water splitting	H_2_: 19.8 μmol g^–1^h^–1^	AQE = 1.3% (380 nm)	59 (2015)
					O_2_: 10.3 μmol g^–1^h^–1^		

^a)^MB = methylene blue; MO = methyl orange; RhB = rhodamine B; AO7 = acid orange 7;.

^b)^DE = degradation efficiency; CE = conversion efficiency; AQE = apparent quantum efficiency.

The CdS/Au/TiO_2_ system, which was fabricated using a simple photochemical technique, is the first example of an all‐solid‐state Z‐scheme system.^26^ Under UV irradiation, the photoexcited electrons in the CB of TiO_2_ transferred to Au and then to the VB of CdS, subsequently recombined with the holes photogenerated in CdS. Simultaneously, the photogenerated electrons in the CB of CdS and the holes in the VB of TiO_2_ exhibited a strong reduction power and oxidation power, respectively, leading to a high photocatalytic reduction of methylviologen (MV^2+^). Moreover, the photoexcited holes in the VB of CdS were recombined with electrons from TiO_2_, resulting in improvement in the photostability of CdS. Similarly, the photocatalytic activities of CdS/Au/ZnO and CdS/Au/TiO_1.96_C_0.04_ were also improved due to the solid‐state Z‐scheme mechanism.^47^, ^49^

Recently, Ag nanoparticles have been extensively investigated as solid electron mediator for the design of a high‐efficiency Z‐scheme system. For the visible‐light‐driven Z‐scheme of Ag_3_PO_4_/Ag/SiC, the band gap energies of Ag_3_PO_4_ and SiC were approximately 2.41 and 2.58 eV, respectively.^50^ Under visible‐light irradiation (*λ* ≥ 420 nm), the photogenerated electrons and holes appeared in both Ag_3_PO_4_ and SiC. The photoinduced electron in the CB of Ag_3_PO_4_ easily shifted to the Ag nanoparticles and recombined with the photogenerated holes in the VB of SiC, resulting in a high efficiency of the electron‐hole separation and an enhancement in the photocatalytic activities. The solid‐state Z‐scheme system of ZnRh_2_O_4_/Ag/Ag_1–_ *_x_*SbO_3–_ *_y_* was developed to split pure water under visible light irradiation.^51^ In this system, Ag acted as a solid‐state electron mediator for the transfer of electrons from the conduction band of Ag_1–_ *_x_*SbO_3–_ *_y_* to the valence band of ZnRh_2_O_4_. As a result, the water‐splitting activity of ZnRh_2_O_4_/Ag/Ag_1–_ *_x_*SbO_3–_ *_y_* was higher than that of ZnRh_2_O_4_, Ag_1–_ *_x_*SbO_3–_ *_y_*, and ZnRh_2_O_4_/Ag_1–_ *_x_*SbO_3–_ *_y_*. With Ag nanoparticles as the solid electron mediator, other Z‐scheme systems, such as Ag_2_CO_3_/Ag/AgBr, Ag_3_PO_4_/Ag/SiC, and α/β‐Bi_2_O_3_/Ag/AgCl, have also been reported enhancement in their photocatalytic activities.^50^, ^52^, ^53^

Notably, noble‐metal nanoparticles (Au, Ag) used as photosensitizers can strongly absorb visible light due to their localized SPR effects.^51^, ^54^, ^55^ For the visible‐light‐driven plasmonic Z‐scheme system of H_2_WO_4_/Ag/AgCl reported by Yu et al.,^54^ AgCl (E_g_ = 3.25 eV) was not photoexcited under visible‐light irradiation (*λ* ≥ 420 nm); however, H_2_WO_4_ (E_g_ = 2.48 eV) absorbed visible‐light photons to produce photogenerated electrons and holes. Meanwhile, photoinduced electrons and holes appeared in the Ag nanoparticles because of the SPR effects. The photogenerated electrons of H_2_WO_4_ transferred to the Ag nanoparticles to recombine with the plasmon‐induced holes. Simultaneously, the holes of H_2_WO_4_ oxidized organic substances. However, the plasmon‐induced electrons of the Ag nanoparticles were injected into the CB of AgCl to reduce oxygen. Other visible‐light‐driven plasmonic Z‐scheme systems, including Ag/AgCl/Bi_20_TiO_32_, α‐β‐Bi_2_O_3_/Ag/AgCl, Ag/AgCl/ZnO, and Ag/AgCl/BiOCl, have also been investigated.^53^, ^55^, ^56^ In these visible‐light‐driven plasmonic Z‐scheme systems, PS II or PS I is photoexcited, and the SPR effect of the Ag nanoparticles became significant for the Z‐scheme photocatalytic activities.^53^, ^54^, ^55^, ^57^ However, when both PS II and PS I are photoexcited, Ag nanoparticles primarily act as the electron mediator.^50^, ^52^, ^57^, ^58^

In addition to the noble metals discussed above, some low‐cost nonmetal materials and metal oxides with excellent conductivities can also be used as electron mediators in solid state Z‐scheme systems. RGO was employed as a solid‐state electron mediator to promote electron transfer between the photocatalyst particles in the Z‐scheme system.^27^, ^48^, ^59^, ^60^, ^61^ Kudo et al. reported a solid state Z‐scheme system (BiVO_4_)/RGO/(Ru/SrTiO_3_:Rh) for water splitting.^61^ Under visible light irradiation, the RGO provides pathways for the photogenerated electrons in BiVO_4_ and the holes in Ru/SrTiO_3_:Rh to recombine, leaving holes in BiVO_4_ and electrons in Ru/SrTiO_3_:Rh to split the water. This “Z” mechanism of electron flow enhanced the charge separation efficiency, resulting in enhancement of the photocatalytic activities. Recently, Kudo et al. also demonstrated that RGO was used to construct Z‐scheme systems that consist of various metal sulphides.^59^ The Z‐scheme system composed of CuGaS_2_ and RGO‐TiO_2_ continuously splits water into H_2_ and O_2_ for 12 h, producing a 1.3% apparent quantum yield under 380 nm of monochromatic light irradiation. Our group fabricated a Fe_2_V_4_O_13_/RGO/CdS Z‐scheme system that perpendicularly grew on a stainless‐steel mesh for the photocatalytic reduction of CO_2_ into methane.^48^ The advantage over the precedent powder Z‐scheme systems is the presence of an “artificial lawn” that may provide a suggestive model for designing an integrated system for practical applications. Additionally, indium–tin oxide (ITO), W, and Cd may also be suitable for the construction of CaFe_2_O_4_/ITO/WO_3_, WO_3_/W/PbBi_2_Nb_1.9_Ti_0.1_O_9_, and ZnO/Cd/CdS Z‐scheme systems, respectively. The enhanced the photocatalytic activity of these systems is attributed to the fast recombination of photogenerated holes in PS I and electrons in PS II within the conductive support.^8^, ^62^, ^63^

## 5 Summary and Perspectives

Photocatalysis is a promising avenue for solving environmental and energy issues in the future, which is attracting an ever‐growing number of scientists to this field. Thus far, the development of photocatalysts with high efficiency, long‐term stability, and at a low cost is the main challenge for their practical application, and it is difficult for a single‐component photocatalyst to simultaneously possess all of these features. The formation of Z‐scheme photocatalytic systems effectively expands the utilization of visible light, improves the separation/transportation of the charge carriers, and substantially enhances the efficiency of the photocatalytic activities.

Z‐scheme systems with shuttle redox mediators are primarily used for efficient water splitting. In this system, the effective separation of charge carriers occurs via recyclable redox reactions of the shuttle redox mediators. However, Z‐scheme systems with shuttle redox mediators have various negative effects, such as back reactions, visible light absorption of the redox mediators and short‐term stability. Z‐scheme systems with solid‐state electron mediators typically suppress the above shortcomings because of the absence of redox mediators. Solid‐state electron mediators provide a pathway for the photogenerated electrons in PS II and the holes in PS I to quickly travel and recombine, leaving more electrons in PS I and more holes in PS II to participate in the redox reaction. Additionally, the SPR effect of metal nanoparticles (Au, Ag) also plays an important role in Z‐scheme systems. When PS II or PS I is photoexcited, the SPR effect of metal nanoparticles becomes significant for Z‐scheme photocatalytic activities. However, when both PS II and PS I are photoexcited, the metal nanoparticles mainly act as electron mediators. The contact interface of PS I–PS II has a significant impact on the charge transfer in the direct Z‐scheme system without electron mediators. A high‐quality interface of PS I–PS II facilitates the transfer of photogenerated charge carriers across the interface, resulting in an enhancement of the photocatalytic performance. As the increasing amount of literatures presented direct Z‐scheme system, it must be strict and careful for distinguishing the direct Z‐scheme system from traditional heterostructure systems such as type‐II heterostructure. Recently, our group applied transient time‐resolved luminescence decay measurements to analyze the difference between type‐II heterostructure and direct Z‐scheme system.^27^ Moreover, Li's group applied spatially resolved surface photovoltage spectroscopy (SRSPS) to obtain direct evidence of highly anisotropic photogenerated charge separation on different facets of a single BiVO_4_ photocatalyst.^98^ This in situ technique may also be applicable to detect the charge transfer process in direct Z‐scheme system.^28^

Great progress has been achieved in the investigation of Z‐scheme systems. However, the photocatalytic reaction is a complex process, and there are many important aspects that need further research, including developing new photocatalytic materials, fundamentally understanding the separation and transport of charge carriers on the interface, and understanding photocatalytic reaction pathways. Therefore, a greater knowledge of the Z‐scheme photocatalytic mechanism and the exploration of new materials are indispensable for making substantial breakthroughs for the practical application of photocatalysts. In this regard, several key considerations could be concerned, including: i) a deep understanding of processes, such as the charge carrier transfer processes and the photocatalytic reaction pathways. The research would integrate experimental and computational approaches to evaluate photocatalytic activity, and would advance fundamental understanding of Z‐scheme photocatalytic mechanism. The transient time‐resolved luminescence decay measurements are well applied to analyze the behavior of photogenerated charge carriers, due to the high time resolution. Surface photovoltaic technique is another advanced technology for probing into the charge carrier transfer processes. ii) The shuttle redox mediators or solid‐state electron mediators of Z‐scheme system plays an important role in charge carrier transportation. It is urgent to develop new shuttle redox mediators or solid‐state electron mediators for solving unfortunate problem, such as backward reactions and light absorption of mediators. iii) Exploitation of new Z‐scheme photocatalytic system is also desired to avoid the mentioned problems in the inorganic Z‐scheme photocatalytic system. A hybrid Z‐scheme by integrating BiVO_4_ and a platinized protein photosystem I (PSI) in an all‐solid‐state was constructed for H_2_ evolution without redox mediators. This hybrid system provides a new means of using a photosynthetic protein as a practical material in the design of a photocatalytic system.^99^

Although the conversion efficiency is still low at the present stage, the Z‐scheme photocatalytic system that mimics the natural photosynthesis in green plants may be the most promising photocatalytic system in photocatalytic field. It is sincerely expected that multiple collaboration for this rapidly evolving field can lead to a breakthrough in the efficiency for the commercialization and industrialization.
